# Enhancing the Permeate Flux Improvement of Direct Contact Membrane Distillation Modules with Inserted S-Ribs Carbon-Fiber Filaments

**DOI:** 10.3390/membranes14050098

**Published:** 2024-04-25

**Authors:** Chii-Dong Ho, Yi-Wun Wang, Yi Chao, Thiam Leng Chew, Ming-Shen Jiang, Jian-Har Chen, Ching-Yu Li

**Affiliations:** 1 Department of Chemical and Materials Engineering, Tamkang University, Tamsui, New Taipei 251301, Taiwan; ywwangi@mail.tku.edu.tw (Y.-W.W.); 612400498@o365.tku.edu.tw (M.-S.J.); 612400571@o365.tku.edu.tw (J.-H.C.); 612400464@o365.tku.edu.tw (C.-Y.L.); 2 Department of Chemical Engineering, Faculty of Engineering, Universiti Teknologi Petronas, Seri Iskandar 32610, Perak, Malaysia; thiamleng.chew@utp.edu.my; 3 CO2 Research Center (CO2RES), Institute of Contaminant Management, Universiti Teknologi Petronas, Seri Iskandar 32610, Perak, Malaysia

**Keywords:** DCMD module, permeate flux, S-ribs filament, turbulence promoter, flow pattern

## Abstract

Three widths of manufacturing S-ribs carbon-fiber filaments acting as turbulence promoters were implemented into the flow channel of direct contact membrane distillation (DCMD) modules to augment the permeate flux improvement in the present study. Attempts to reduce the disadvantageous temperature polarization effect were made by inserting S-ribs turbulence promoters in improving pure water productivity, in which both heat- and mass-transfer boundary layers were diminished due to creating vortices in the flow pattern and increasing turbulence intensity. The temperature polarization coefficient $t_{temp}$ was studied and found to enhance device performance (less thermal resistance) under inserting various S-ribs carbon-fiber thicknesses and operating both cocurrent- and countercurrent-flow patterns. The permeate fluxes in the DCMD modules with inserted S-ribs carbon-fiber turbulence promoters were investigated theoretically by developing the mathematical modeling equations and were conducted experimentally with various design and operating parameters. The theoretical predictions and experimental results exhibited a great potential to considerably achieve permeate flux enhancement in the new design of the DCMD system. The DCMD module with inserted S-ribs carbon-fiber turbulence promoters in the flow channel could provide a relative permeate flux enhancement up to 37.77% under countercurrent-flow operations in comparisons with the module of using the empty channel. An economic consideration on both permeate flux enhancement and power consumption increment for the module with inserted S-ribs carbon-fiber filaments was also delineated.

## 1. Introduction

Membrane distillation (MD) has gained significant attraction in both industrial and academic settings in recent years due to its technical feasibility. The advancements in separation and water purification techniques have focused on high permeate flux and low energy consumption, particularly in the areas of membrane materials, module fabrication, transport phenomena, and fouling mitigation. MD [1,2] has gained attractive attention due to the existing thermal gradients built up by both microporous hydrophobic membrane surfaces, which are always in contact with both hot and cold bulk solutions at their saturation points to allow the passage of vapors only [3,4]. The MD process involving both heat and mass transfer undergoes temperature and concentration polarization effects [5] near the membrane surface, which diminish the device performance. A higher temperature causes a larger impact on the temperature polarization effect [6], while a higher feed composition induces a more pronounced influence on the concentration polarization effect [7]. The significant thermal resistance across the membrane with a considerable removal of the latent heat associated to water evaporation occurs on the thermal boundary layer due to the vapor pressure gradient across the hydrophobic membrane, which causes the feed temperature decrement, called as the temperature polarization effect [8], and consequently, the net thermal driving force between the bulk temperature and membrane surface temperature to transport the permeate flux thus declines [9].

Augmentation of the temperature driving-force gradient by implementing turbulence promoters to suppress the boundary layer between the bulk and membrane surface results in the increment of transmembrane permeate flux by alleviating the temperature polarization effect [10]. Incorporating suitable flow amendment configurations of turbulence promoters to amplify the hydrodynamic conditions has proven to be beneficial for the simultaneous mass and heat transfer in MD in the boundary layer, such as eddy promoters [11,12] and flow deflectors [13,14,15]. Moreover, the development of advanced composite membranes [16,17] meets the requirements of an ideal membrane with a lower conductive heat loss and a higher mass-transfer rate. Many previous studies focused on the eddies and wakes owing to vortices and secondary flows [18] as the hot stream passes through the spacer strands and proposed the mathematical model to predict heat- and mass-transfer enhancements in the MD desalination system with respect to traditional membrane modules without inserting turbulence promoters [19]. The mathematical heat and mass transport modeling approaches applied in membrane distillation were reviewed [20] and simulated using computational fluid dynamics (CFD) [12,21].

Various approaches, such as the use of spacers [22], filaments [23], and roughened surfaces [24], have been proposed to improve the hydrodynamic conditions by suppressing the thermal boundary layer, thereby enhancing the heat- and mass-transfer rates. The influence affects both heat-transfer rates and heat-transfer coefficients [25,26] which were recognized to identify the relative significance of each heat-transfer mechanism. All of the spacers, filaments, and roughened surfaces play an important role as a turbulence promoter in both heat and mass transport, and thus, heat-transfer improvement is achieved. In MD modules, permeate is transported with simultaneous heat transfer, resulting in complex heat-transfer mechanisms by the temperature difference between the hot and cold feed streams. The resulting turbulence reduces the temperature polarization effect, leading to improved heat and mass-transfer rates [10]. However, the heat- and mass-transfer rate enhancement are at the expense of the pressure drops with power consumption increments due to the augmented turbulent intensity induced by the turbulence promoters. To assess the economic and technical viability, the ratio of transfer rate enhancement to energy consumption increment was evaluated [27]. A recent study has demonstrated the effectiveness of a segment design involving the machining of a carbon-fiber sheet with various geometric shapes to increase turbulent intensity and alleviate the temperature polarization effect in the flow channel of DCMD modules. This disruption of the thermal boundary layer has been shown to significantly increase the convective heat-transfer coefficient by introducing carbon-fiber turbulence promoters into the hot feed stream, thereby changing the flow direction from the curved-shape grid to the grid, as compared to modules with feed channels lacking carbon-fiber filaments. However, the benefits of using turbulence promoters in DCMD are weighed down by their disadvantages associated with the increment of the pressure drop in the feed stream [28].

The present study actually extends the existing study except for inserting S-ribs carved-shape carbon-fiber filaments instead of using straight-line carbon-fiber filaments [29] to accomplish the augmented turbulent intensity due to changing the flow direction by the hydrodynamic angle. This work is based on the principle of the introduction of S-ribs carbon-fiber turbulence promoters (1 mm thickness) with various widths (3 mm, 4 mm, and 5 mm) as feed channel filaments into the DCMD module, which would suppress the boundary layer between the bulk and membrane surface to achieve a smooth transition for the turbulent flow near the membrane surface of fluid flow due to the increased turbulence intensity and decreased thermal boundary layer resistance. Two strata of microscopic and plug-flow descriptions are used to represent the real processes by mathematics, which evolve from transport phenomena principles. These two descriptions are depicted to be related to the complexity of the heat-transfer mechanism in the present DCMD system and conjugated with the dusty-gas model to describe the essential membrane coefficient models, which can estimate the permeate flux across the microporous hydrophobic membrane. In this study, we develop a theoretical model for the DCMD module to estimate the module’s permeate fluxes and analyze its trade-off considerations for energy consumption. The primary objective is to fabricate S-ribs turbulence promoters and insert them into the hot stream channel to mitigate its temperature polarization effect, ultimately leading to an increase in vapor permeate flux. Additionally, we refine and validate a regression equation for the heat-transfer enhancement factor through experimental verification.

## 2. Experimental Setup and Materials

**Figure 1:**
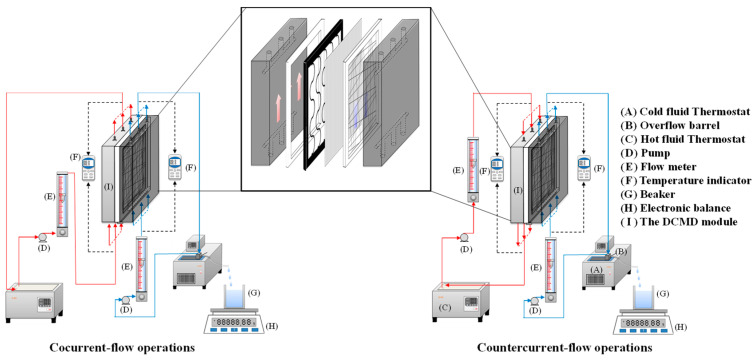
Schematic diagram of fabrication structure and experimental setup for the DCMD system of the S-ribs carbon-fiber-filled DCMD module.

**Figure 2:**
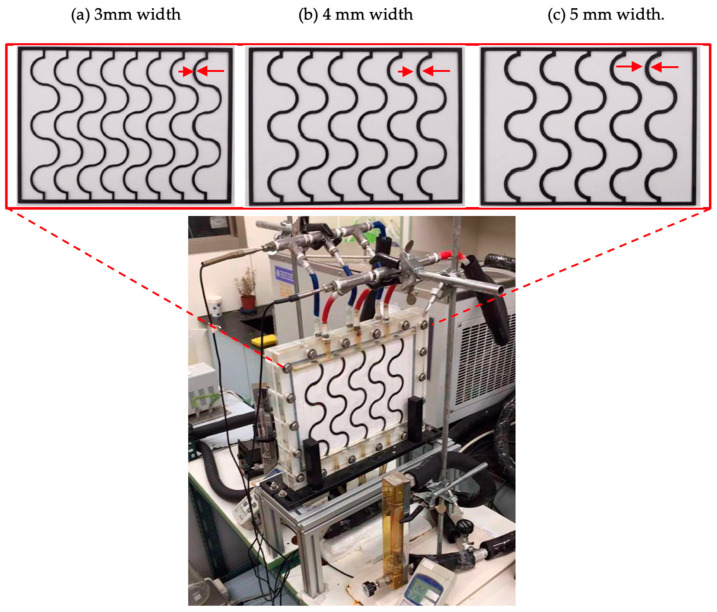
A photo of the experimental setup of three widths of S-ribs carbon-fiber filaments.

The carbon fiber used in this study was chosen concerning its lower cost, mechanical strength to preventing membrane vibration, and easy fabrication of the various geometric shapes of the filaments. In addition, incorporating the mesh design (say S-ribs carbon fiber) into the DCMD module was proposed so as to act as turbulence promoters and amplify the hydrodynamic conditions. The fabrication details of S-ribs carbon-fiber filaments and the schematic configuration are depicted in Figure 1. Figure 2 showcases a photograph of the flat-plate DCMD experimental setup, featuring acrylic plates as external walls within a parallel-plate channel. S-ribs carbon-fiber sheets, cut in an S-shape with a 1 mm thickness, serve as turbulence promoters inserted into the hot saline feed stream to induce eddy motion. The S-ribs carbon-fiber filaments adhered to the membrane surface in the hot feed side were inserted in parallel vertically into a parallel-plate channel to conduct a two-stream operation. Meanwhile, the cold feed side was constructed using a 0.1 mm nylon fiber wound as a supportive grid to prevent membrane vibration and wrinkling. Both supporting materials provide mechanical strength to prevent membrane vibration and act as turbulence promoters.

The experimental setup involves two parallel-plate parallel channels (*L* = 0.21 m, *W* = 0.29 m, *d* = 2 mm), separated by a hydrophobic composite membrane made of PTFE/PP (polytetrafluoroethylene and polypropylene, J020A330R, ADVANTEC Toyo Roshi Kaisha, Ltd., Tokyo, Japan) with a thermal conductivity of $5.0\times 10^{-4}\;cal/cm\;sec\;{}^{\circ}C$ (All-Fluoro Co., Ltd. Taoyuan, Taiwan) and a water vapor permeability resistance of 4 sec/m [30]. The membrane possesses a nominal pore size of 0.2 µm, a porosity of 0.72, and a thickness of 130 µm, serving as the permeating porous medium in this study. A 2 mm-thick silicon rubber was affixed to the acrylic plate to prevent leakage and create two spacer conduits of 2 mm for each channel, respectively. Figure 2 illustrates the top views of S-ribs carbon-fiber filaments with various widths (3 mm, 4 mm, and 5 mm) as a design parameter. These S-ribs carbon-fiber filaments provide mechanical strength to prevent membrane vibration and act as turbulence promoters. The effective permeate areas were partially obstructed by these three widths of S-ribs carbon-fiber filaments, covering approximately 13% of the hydrophobic membrane, a factor considered in the calculation procedure.

The artificial saline water, consisting of 3.5 wt% NaCl, was prepared by adding inorganic salts (NaCl) to both distilled water and pure water, respectively. This saline solution was pumped (51K40RA-A, ASTK, New Taipei, Taiwan) from the thermostat (G-50 and D650, DENG YNG, New Taipei, Taiwan) at specified temperatures (45 °C, 50 °C, 55 °C, and 60 °C). Inlet and outlet temperatures were measured using thermometer probes (TM-946, Lutron, New Taipei, Taiwan) connected to both sides of the flat-plate membrane modules. The operational conditions of saline feed streams, with various flow rates (0.3, 0.5, 0.7, and 0.9 L/min), were adjusted using flow meters (FE-091312-D, Fong-Jei, Hsinchu, Taiwan) and a controller (N12031501PC-540, Protec, Brooks Instrument, Hatfield, PA, USA), while maintaining the temperature at 25 °C for the cold stream (FN-0423112-F, Fong-Jei, Hsinchu, Taiwan). The conductance of the permeate flux was collected and measured, being less than 1.5 μs/cm. Comparisons were made of permeate fluxes under various operation conditions to assess the device performance between two modules with and without inserting S-ribs carbon-fiber turbulence promoters. The experimental run of the permeate flux was collected and weighed using an electronic balance (XS 4250C, Precisa Gravimetrics AG, Dietikon, Switzerland) for measurement and recording on the PC.

## 3. Theoretical Formulations

### 3.1. Mass and Heat Transfer

**Figure 3:**
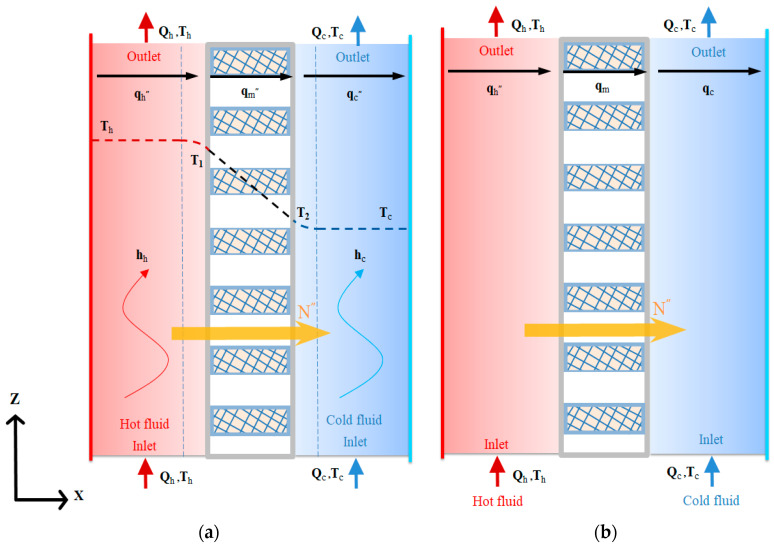
Microscopic and plug-flow descriptions of mass- and heat-transfer mechanisms. (**a**) Microscopic description; (**b**) Plug-flow description. (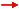
Hot fluid; 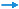
Cold fluid; 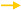
Permeate flux; 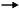
Heat flux).

Theoretical statements combining both heat- and mass-transfer mechanisms were formulated for the DCMD system with microscopic and plug-flow descriptions, as shown in Figure 3a,b, respectively. Figure 3a,b are two models that evolve from transport phenomena principles. Two levels of microscopic and plug-flow descriptions used to represent real processes by mathematics. These two levels are depicted to be related to the complexity of the heat-transfer mechanism in the present DCMD system. The microscopic description involves a phenomenological approach, representing the system as a continuum, while the plug-flow description deals with only the largest component of the gradient in the balance equation by the bulk flow, neglecting all diffusion terms. A mass-transfer model, coupled with heat-transfer behavior, was developed to illustrate the concentration gradient of the hot saline feedwater, leading to the vapor diffusing exclusively through the porous hydrophobic membrane and condensing in the cold stream, completing the distillation process. This occurs due to the temperature gradients at both membrane surfaces. The theoretical analysis of the DCMD aims to elucidate how the saline water is initially vaporized and eventually condensed at the pore entrances of both membrane surfaces. This is achieved through the permeate/water equilibrium governed by vapor diffusion and enthalpy flow conservation through heat conduction, as detailed below.

The membrane permeation coefficient ($c_{m}$) and the trans-membrane saturation vapor pressure difference (ΔP) were evaluated to determine the permeate flux for membrane distillation processes [31,32].

(1) $$N^{\prime \prime }=c_{m}\Delta P=c_{m}\left[P_{1}^{sat}(T_{1})-P_{2}^{sat}(T_{2})\right]\;\;\;\;\;{=c}_{m}{\left.\frac{dP}{dT}\right|}_{T_{m}}\left(T_{1}-T_{2}\right)=c_{m}\frac{P_{m}\lambda M_{w}}{RT_{m}^{2}}\left(T_{1}-T_{2}\right)$$

The dusty-gas model was used to describe three essential membrane coefficient models based on a comparison between the mean free path of vapor molecules and the membrane pore sizes, which can estimate the mass flux across the microporous hydrophobic membrane. The influence of the Poiseuille flow can be neglected [33] when the pore size of the membrane is relatively small. Considering that a membrane of 0.2 µm is used in the present study, we may conclude that the addition of the Knudsen and molecular diffusion models is appropriate to estimate the permeate flux through the membrane. The combination of the Knudsen diffusion model (due to the smaller mean free path of vapor molecules than the membrane pore size) and the molecular diffusion model (due to the concentration gradient across the membrane) was investigated [34] to dominate the mass-transfer mechanism and presented by an equation of the membrane permeation coefficient for deaerated microporous membranes to predict the permeate flux through the membrane. The membrane permeation coefficient is the combination of the Knudsen diffusion model and molecular diffusion model as follows:

(2) $$c_{m}={\left(\frac{1}{c_{K}}+\frac{1}{c_{M}}\right)}^{-1}={\left\{{\left[\frac{2\sqrt{8\pi }}{3}\frac{\varepsilon \;r}{\tau \delta_{m}}{\left(\frac{M_{w}}{RT_{m}}\right)}^{1/2}\right]}^{-1}+{\left[{\left|Y_{m}\right|}_{ln}\frac{D_{m}\varepsilon }{\delta_{m}\tau }\frac{M_{w}}{RT_{m}}\right]}^{-1}\right\}}^{-1}$$

The temperature differences between the streams near the membrane surfaces and those of the bulk feed streams are utilized to estimate the temperature polarization coefficient. Predictions are made regarding the temperature gradients of membrane surfaces on both the feed stream and permeate side across the entire module, influencing heat transfer. Consequently, the permeate flux is calculated using the mass-transfer modeling equation presented in Equation (1). The saturation vapor pressure ($P_{1}^{sat}$) of the hot saline feed side is estimated using the water activity coefficient ($a_{w}$), determined through a correlation [1]:

(3) $$P_{1}^{sat}=x_{w}a_{w}P_{w}^{sat}$$

(4) $$a_{w}=1-0.5x_{\mathrm{NaCl}}-10x_{\mathrm{NaCl}}^{2}$$

and the tortuosity (τ) can be estimated using the porosity of the membrane [35]

(5) τ=1/ε

**Figure 4:**
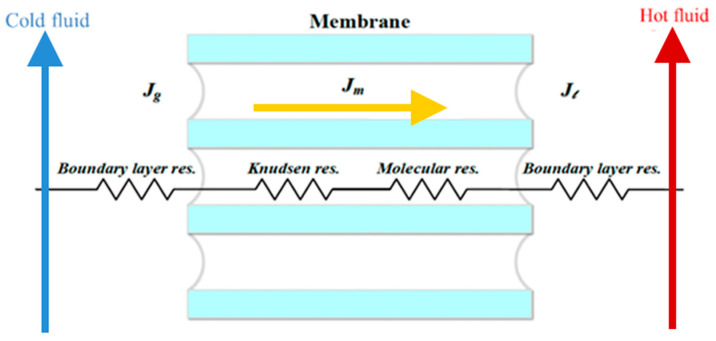
Schematic mass-transfer resistances for three mass-transfer regions of membrane contactor. (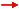
Hot fluid; 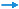
Cold fluid; 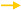
Permeate flux).

According to the macroscopic description of the temperature gradient of the DCMD module in Figure 3a, the heat balances of enthalpy flow conservation in the non-isothermal process were made within each heat-transfer region in the DCMD module as follows: (a) the hot saline water stream; (b) the hydrophobic composite membrane; and (c) the cooling water stream. Vapor flux permeating through the hot feed stream, microporous hydrophobic membrane and cold stream for the modules with/without inserted S-ribs carbon-fiber turbulence promoters was expressed in terms of heat-transfer resistances in series due to the temperature gradient, as shown in Figure 4.

The energy balance equations may be derived by balancing energy under the steady-state operation in both feed channels to and from the membrane surfaces. Equating the amount of heat flux by the conservation law among three regions, one may obtain the following:

(6) $$q_{h}^{\prime \prime }=h_{h}(T_{h}-T_{1})\;\mathrm{the}\;\mathrm{hot}\;\mathrm{saline}\;\mathrm{water}\;\mathrm{feed}\;\mathrm{region}$$

(7) $$q_{m}^{\prime \prime }=N\prime \prime \lambda +\frac{k_{m}}{\delta_{m}}\;\;(T_{1}-T_{2})\;\mathrm{the}\;\mathrm{membrane}\;\mathrm{region}$$

(8) $$q_{c}^{\prime \prime }=h_{c}(T_{2}-T_{c})\;\mathrm{the}\;\mathrm{cooling}\;\mathrm{water}\;\mathrm{region}$$

where N″λ is categorized as the latent heat of vaporization and $k_{m}/\delta_{m}=h_{h}(T_{1}-T_{2})$ is the heat conduction, and the thermal conductivity of the membrane $k_{m}\;$can be determined by the thermal conductivities of vapor in the membrane pore $k_{g}$ and the solid membrane material $k_{s}$ by Warner [36] as

(9) $$k_{m}=\varepsilon \;k_{g}+\left(1-\varepsilon \right)k_{s}$$

Equation (7) can be rewritten by using Equation (1) in terms of the overall heat-transfer coefficient of the membrane Hm including the latent heat across the membrane, which is implicitly related to the temperature polarization coefficient as follows:

(10) $$\begin{array}{ll} q_{m}^{\prime \prime } & =N\prime \prime \lambda +\frac{k_{m}}{\delta_{m}}\;\;(T_{1}-T_{2})\\ & =\left\{c_{m}\frac{\left[\left(1-x_{\mathrm{NaCl}}\right)\left(1-0.5x_{\mathrm{NaCl}}-10x_{\mathrm{NaCl}}^{2}\right)P_{2}+P_{1}\right]\lambda^{2}M_{w}}{2RT_{m}^{2}}+\frac{k_{m}}{\delta_{m}}\right\}\left(T_{1}-T_{2}\right)\\ & =H_{m}(T_{1}-T_{2}) \end{array}$$

### 3.2. Temperature Polarization Coefficient

**Figure 5:**
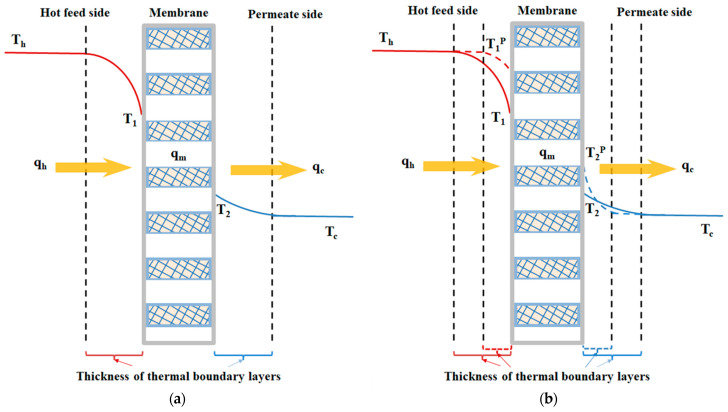
Reduction in thermal boundary polarization layers in the DCMD module. (**a**) Empty channel; (**b**) Channel with inserted turbulence promoters.

The temperature polarization coefficient $\tau_{temp}$ is an indicator to reveal the extent of the thermal boundary-layer resistance between both the hot saline and cooling feed streams, as indicated in Figure 5a,b, which controls the permeate flux through the membrane, and $\tau_{temp}$ is commonly defined as follows:

(11) $$\tau_{temp}=(T_{1}-T_{2})/(T_{h}-T_{c})$$

Actually, inserting the S-ribs carbon-fiber filament in the hot saline water compartment would increase $h_{h}$ (for the module without promoter insertion) to $h_{h}^{p}$ (for the module with promoter insertion) but $h_{c}\;$does not change. According to Equations (6)~(8) and (10), all heat-transfer regions of the microscopic description under steady-state operations with assuming $q^{\prime \prime }=q_{h}^{\prime \prime }=q_{m}^{\prime \prime }=q_{c}^{\prime \prime }$ were developed and illustrated by the schematic diagram in Figure 3a.

(12) $$q^{\prime \prime }=h_{h}\left(T_{h}-T_{1}\;\right)=H_{m}\;(T_{1}-T_{2})=h_{c}(T_{2}-T_{c})$$

or

(13) $$q^{\prime \prime }=\frac{\left(T_{h}-T_{c}\right)}{\frac{1}{h_{h}}+\frac{1}{H_{m}}+\frac{1}{h_{c}}}=h_{c}\left(T_{2}-T_{c}\;\right)=q_{c}^{\prime \prime }$$

Equation (13) can be rewritten as

(14) $$T_{2}-T_{c}=\frac{\left(T_{h}-T_{c}\right)}{\frac{h_{c}}{h_{h}}+\frac{h_{c}}{H_{m}}+1}$$

Similarly, we have the increased value of the temperature gradient (say $T_{2}^{p}-T_{c}$) by using the same performed procedure of Equation (14) in the cold feed region for $h_{h}^{p}>h_{h}$ due to inserting the turbulence promoter, as shown in Figure 5b.

(15) $$\frac{\left(T_{h}-T_{c}\right)}{\frac{h_{c}}{h_{h}^{p}}+\frac{h_{c}}{H_{m}}+1}=T_{2}^{p}-T_{c}>T_{2}-T_{c}$$

Meanwhile, the temperature polarization coefficient of Equation (11) increases with the promoter insertion, i.e.,

(16) $$\frac{\left(T_{h}-T_{c}\right)}{\frac{H_{m}}{h_{h}^{p}}+1+\frac{H_{m}}{h_{c}}}=T_{1}^{p}-T_{2}^{p}=∆T^{p}>{∆T=T}_{1}-T_{2}=\frac{\left(T_{h}-T_{c}\right)}{\frac{H_{m}}{h_{h}}+1+\frac{H_{m}}{h_{c}}}$$

The temperature polarization effect was reduced, as demonstrated in Figure 5b, due to the DCMD module with inserted S-ribs carbon-fiber turbulence promoters, which resulted in the temperature difference enlargement of both membrane surface temperatures ${∆T^{p}(T}_{1}^{p}-T_{2}^{p})>{∆T(T}_{1}-T_{2}$). The gradient of ∆T increases to a higher driving force $∆T^{p}$ with inserted S-ribs carbon-fiber turbulence promoters, and thus, the vapor flux is increased, as shown in Figure 5b. The S-ribs carbon-fiber filaments have been validated to disrupt the laminar boundary layer and intensify vortices or secondary flow characteristics on the membrane surface, responding to changing hydrodynamic conditions. This disruption plays a crucial role in mitigating the temperature polarization effect and enhancing a higher permeate flux.

The alternative expression of Equation (11) was obtained by equating the energy conservation of heat fluxes in each region, say Equations (6) and (10) ($q_{h}^{\prime \prime }=q_{m}^{\prime \prime }$) and Equations (8) and (10) ($q_{m}^{\prime \prime }=q_{c}^{\prime \prime }$), respectively, to replace both membrane surface temperatures ($T_{1}$ and $T_{2}$) in terms of the heat-transfer coefficients ($h_{h}$, $h_{c}$ and $H_{m}$) as follows:

(17) $$T_{h}=T_{1}+\frac{H_{m}}{h_{h}}(T_{1}-T_{2})$$

(18) $$T_{c}=T_{2}-\frac{H_{m}}{h_{c}}\left(T_{1}-T_{2}\right)$$

Then, a more simplified form of $\tau_{temp}$ in Equation (11) can be rewritten in terms of the heat-transfer coefficient as

(19) $$\tau_{temp}=\frac{h_{h}h_{c}}{h_{h}h_{c}+h_{h}H_{m}+h_{c}H_{m}}$$

The procedure for calculating theoretical predictions of the mass-transfer coefficient was performed by continuously iterating $T_{1}$ and $T_{2}$ from Equations (17) and (18) within the convergence tolerance. The calculated convective heat-transfer coefficients were calculated from Equation (1) and validated by the experimental results, delivered to predict theoretically not only in the hot/cold bulk flows ($T_{h}$ and $T_{c}$) but also those on the membrane surfaces ($T_{1}$ and $T_{2}$) of both hot and cold feed streams, respectively.

### 3.3. Governing Equations by Macroscopic Modeling

**Figure 6:**
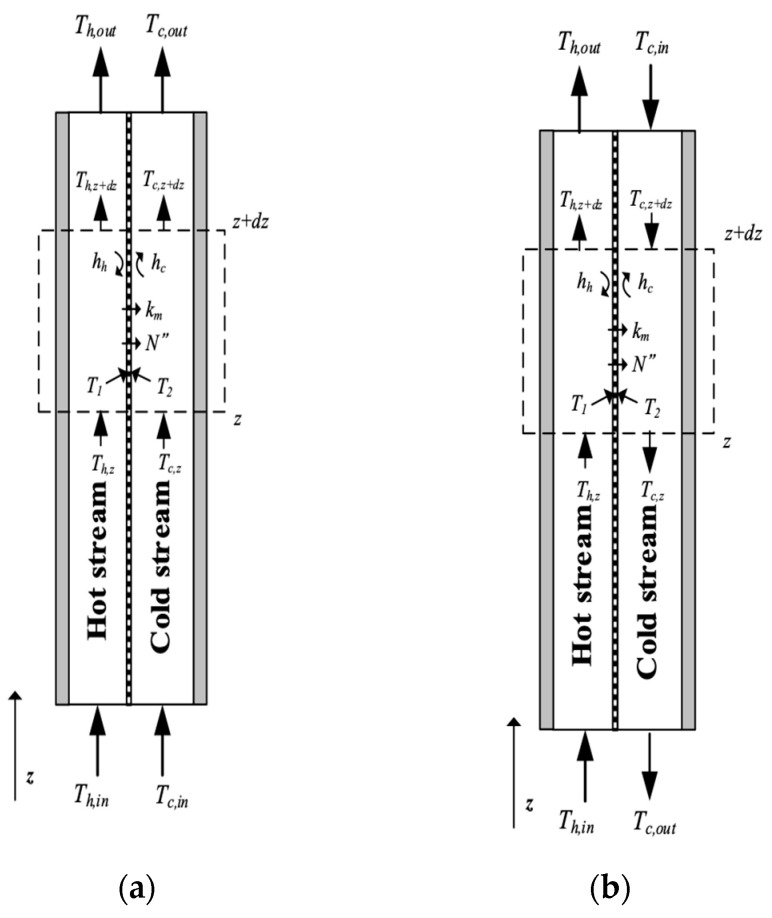
The energy balance made within a finite fluid element. (**a**) Cocurrent-flow operations; (**b**) Countercurrent-flow operations.

Both temperature variations in both hot saline and cooling feed streams along the flow direction were developed, as illustrated in Figure 6, by energy conservation in one-dimensional governing equations according to the macroscopic modeling to solve the temperature distributions of both streams in terms of the temperature polarization coefficient $\tau_{temp}$ as

(20) $$\frac{dT_{h}}{dz}=\frac{-q^{\prime \prime }W}{Q_{h}\;\rho_{h}C_{p,h}}=\frac{-W}{Q_{h}\;\rho_{h}C_{p,h}}H_{m}\tau_{temp}(T_{h}-T_{c})$$

(21) $$\frac{dT_{c}}{dz}=\frac{q^{\prime \prime }W}{Q_{c}\;\rho_{c}C_{p,c}}=\frac{W}{Q_{c}\;\rho_{c}C_{p,c}}H_{m}\tau_{temp}(T_{h}-T_{c})\;\;\mathrm{Cocurrent-flow}\;\mathrm{operations}$$

(22) $$\frac{dT_{c}}{dz}=\frac{-q^{\prime \prime }W}{Q_{c}\;\rho_{c}C_{p,c}}=\frac{-W}{Q_{c}\;\rho_{c}C_{p,c}}H_{m}\tau_{temp}(T_{h}-T_{c})\;\mathrm{Countercurrent-flow}\;\mathrm{operations}$$

Temperature fields of both hot and cold feed streams were solved with the use of two simultaneous ordinary differential equations of Equations (20) and (21) for cocurrent-flow operations (or Equations (20) and (22) for countercurrent-flow operations) by marching the 4th Runge-Kutta method along the length of the module, which can quantify the enhanced permeate flux by the turbulence intensity augmentation with inserted S-ribs carbon-fiber turbulence promoters.

### 3.4. Hydraulic Consumption Increment

The hydraulic consumption increment is expected and required due to inserting S-ribs carbon-fiber filaments into the hot saline stream, which may be determined using the Fanning friction factor ($f_{F}$) [37], considering only the friction losses to walls of both hot and cold streams as

(23) $$H=H_{h}+H_{c}=Q_{h}\rho_{h}lw_{f,h}+Q_{c}\rho_{c}lw_{f,c}$$

(24) $$lw_{f,i}=\frac{2f_{F,i}{{\bar{v}}_{i}}^{2}L}{D_{h,i}},i=h,c$$

in which

(25) $${\bar{v}}_{h}=\frac{Q_{h}}{\left(dW-D_{1}W_{1}N_{1}\right)},\;{\bar{v}}_{c}=\frac{Q_{c}}{A}\;$$

(26) $${De}_{h,h}=\frac{4\left(dW-D_{1}W_{1}N_{1}\right)}{2\left(d+W+D_{1}N_{1}\right)}\;,{De}_{h,c}=\frac{4dW}{2\left(d+W\right)}$$

**Figure 7:**
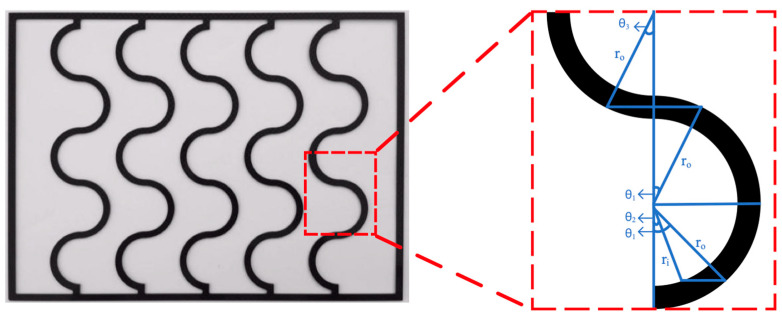
The average carbon-fiber width of S-ribs carbon-fiber turbulence promoters.

The hydraulic equivalent diameter $D_{h,h}$ of modules with embedding S-ribs carbon-fiber turbulence promoters was calculated by the wetted area A and wetted perimeter P as the average carbon-fiber width of S-ribs carbon-fiber turbulence promoters, say 4A/P, as shown in Figure 7, which were evaluated by averaging various sections of the S-rib shape.

The Fanning friction factor can be estimated using a correlation based on the aspect ratio of the channel (α=d/W) [38]:

(27) $$f_{F,h}=\frac{C}{{Re}_{h}},f_{F,c}=\frac{C}{{Re}_{c}}$$

(28) $$C=24\left(1-1.3553\alpha +1.9467\alpha^{2}-1.7012\alpha^{3}+0.9564\alpha^{4}-0.2537\alpha^{5}\right)$$

The relative extent of the hydraulic consumption increment $I_{P}$ was illustrated by calculating the percentage increment in the module with inserted S-ribs carbon-fiber filaments, which was based on the module of using empty channels as

(29) $$I_{p}=\frac{H_{promoter}-H_{empty}}{H_{empty}}\times 100\%$$

### 3.5. Heat-Transfer Enhancement Factor

S-ribs carbon-fiber turbulence promoters were incorporated into the open conduit of the hot saline feed stream, replacing the device of an empty channel. The correlation of heat-transfer coefficients [39] for the module using filament-filled channels was proposed through multiple linear regressions. The heat-transfer enhancement factor $\alpha^{p}$ was introduced [11] to calculate augmented convective heat-transfer coefficients in DCMD modules with inserted S-ribs carbon-fiber filaments, employing an iterative procedure:

(30) $$\alpha^{p}=Nu^{p}/Nu_{lam}$$

where the heat-transfer equivalent diameter ${De}_{h,h}^{h}\;$was defined [40] as follows:

(31) $${De}_{h,h}^{h}=\frac{4\left(dW-D_{1}W_{1}N_{1}\right)}{W-D_{1}N_{1}}$$

(32) $$Nu_{lam}=4.36+\frac{0.036\mathrm{RePr}\left({De}_{h,h}^{h}/L\right)}{1+0.011{\left(\mathrm{RePr}\left({De}_{h,h}^{h}/L\right)\right)}^{0.8}}\;\;\mathrm{Empty}\;\mathrm{channel}\;\mathrm{module}$$

(33) $$Nu^{p}=\frac{h_{h}{De}_{h,h}^{h}}{k}\;\;\mathrm{S-ribs}\;\mathrm{carbon-fiber-filled}\;\mathrm{module}$$

The S-ribs carbon-fiber filaments, acting as eddy promoters within the flow channel, play a vital role and offer a comprehensive interpretation of both heat- and mass-transfer behaviors. This interpretation is based on dimensional analysis using Buckingham’s π theorem, relating to the Nusselt number as:

(34) $$Nu^{p}=f\left(\frac{W_{1}}{{De}_{h,h}^{h}},Re,Pr\right)$$

where $W_{1}$ and ${De}_{h,h}^{h}$ are the carbon-fiber width and heat-transfer equivalent diameter of channels with S-ribs carbon-fiber filaments, respectively.

## 4. Results and Discussions

### 4.1. Lessening Temperature Polarization Effect by Inserting Carbon-Fiber Filaments

**Figure 8:**
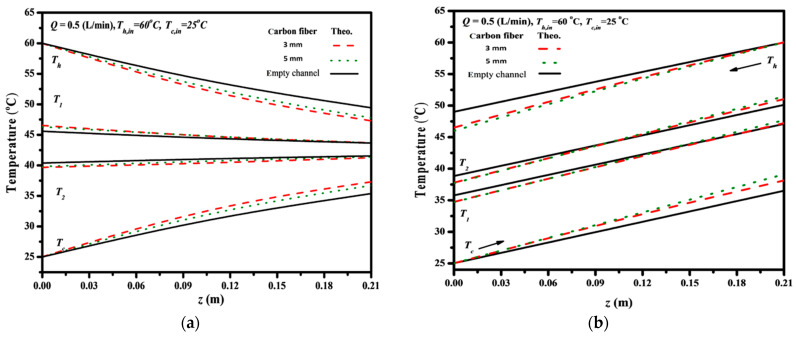
Effects of carbon-fiber widths on temperature distributions along the module. (**a**) Cocurrent-flow operations; (**b**) Countercurrent-flow operations.

Both bulk temperature distributions of hot saline and cold feed streams as well as membrane surface temperatures in DCMD modules were solved numerically using the one-dimensional theoretical model, as presented in Figure 8, along the axial coordinate with the carbon-fiber width as a parameter under both cocurrent- and countercurrent-flow operations, respectively.

In cocurrent-flow operations, theoretical predictions indicate a tapering of the membrane-surface and bulk temperatures along the flowing direction, leading to a reduction in driving-force temperature gradients. Conversely, countercurrent-flow operations maintain a relatively higher average value of the driving-force temperature gradients, resulting in a greater enhancement of permeate flux. The analysis reveals that temperature gradients across both membrane surfaces are higher in flow channels with inserted S-ribs carbon-fiber turbulence promoters compared to those in the empty channel. These increased temperature gradients facilitate greater vapor transport through the membrane, consequently leading to a higher amount of permeate flux condensed in the cooling stream.

**Figure 9:**
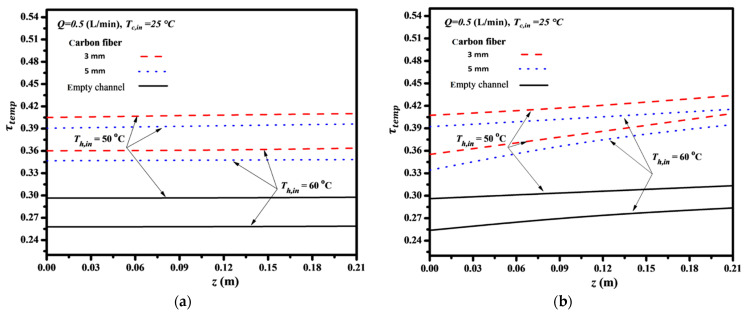
Effects of saline feed temperature and carbon-fiber widths on $\tau_{temp}$. (**a**) Cocurrent-flow operations; (**b**) Countercurrent-flow operations.

Furthermore, theoretical predictions of temperature polarization coefficients $\tau_{temp}$ were determined according to Equation (19) and are presented in Figure 9. The analysis considered the inlet saline temperature and carbon-fiber widths as parameters. The $\tau_{temp}$ increased with decreasing both the carbon-fiber width and the inlet saline temperature for both cocurrent- and countercurrent-flow operations. The descending permeate flux along the flowing channel for cocurrent-flow operations is thus expected in contrast to the almost constant temperature gradient of the countercurrent-flow operations. The larger inlet saline temperature associated with the higher vapor pressure creates the greater permeate flux passing through the membrane and results in the lower temperature difference (say $T_{1}^{p}-T_{2}^{p})$ across the membrane surfaces; therefore, a lower value of $\tau_{temp}$ is achieved. This is because the greater permeate flux transferring through the membrane results in reducing the saturation pressure as well as the water activity coefficient of the hot saline stream. However, comparisons of $\tau_{temp}$ were made on operating the module with inserted S-ribs carbon-fiber filaments of 3 mm and 5 mm with that of the empty channel, as shown in Figure 9. A larger $\tau_{temp}$ was accomplished substantially when the module included inserted S-ribs carbon-fiber filaments due to disturbing the thermal boundary layer on the membrane surface with a smaller thermal resistance. Suppressing the temperature polarization effect results from inserting S-ribs carbon-fiber filaments in the hot saline feed stream to diminish the thermal boundary layer thickness on the membrane surface. Meanwhile, the effect reduction in $\tau_{temp}$ is more significant in countercurrent-flow operations, and thus, a higher $\tau_{temp}$ value is achieved in the countercurrent-flow operation, as demonstrated in Figure 9.

### 4.2. Permeate Flux Enhancement by Inserting S-Ribs Carbon-Fiber Turbulence Promoters

Applying the regressed correlation approach and quantifying the enhanced permeate flux due to inserting S-ribs carbon-fiber turbulence promoters were solved directly through the permeate flux enhancement factor, which was established based on dimensional analysis of using Buckingham’s π theorem in Equation (34). The experimental runs with empty channels and 3 mm, 4 mm, and 5 mm S-ribs carbon-fiber widths were used to determine the correlation for the permeate enhancement factor $\alpha^{p}$, which was expressed in Equation (30) of the Nusselt number as a measure of heat-transfer efficiency as follows:

(35) $$\alpha^{p}=\frac{Nu^{p}}{Nu_{lam}}=1.72(\frac{W_{1}}{{De}_{h,h}^{h}}{)}^{-0.165}{Re}^{-0.04}{Pr}^{0.321}$$

**Figure 10:**
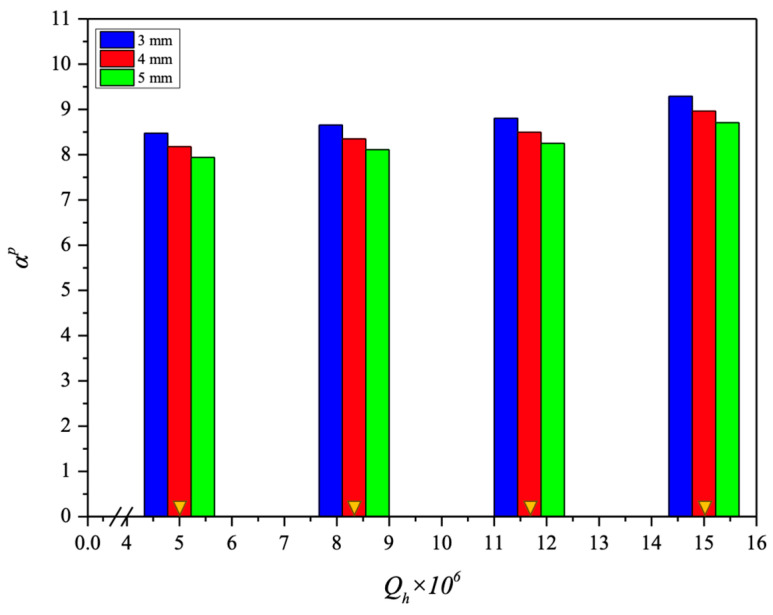
Comparisons of theoretical Nusselt numbers for the channels with inserted S-ribs carbon-fiber turbulence promoters.

The Nusselt number for the empty channel is presented in Equation (32), while the corrected Nusselt number for S-ribs carbon-fiber-filled channels is presented in Equation (33). Furthermore, the correlated Sherwood number was incorporated into the mass-transfer enhancement factor $\alpha^{p}$, as the improved heat-transfer coefficient reduces temperature polarization, and thus the driving force across the membrane is increased. The heat-transfer enhancement factor was derived from the correlation via a regression analysis and expressed in Equation (35) for implementing S-ribs carbon-fiber turbulence promoters, which results in the augmented convective heat-transfer coefficients in membrane distillation modules and presented in Equation (35) as well as in Figure 10. A regression analysis was set up the normal equations for the least square parameters to obtain the correlated equation and the squared correlation coefficient ($R^{2}$) is 0.94. It is concluded from Figure 10 that these corrected Nusselt numbers are more significant in the modules with inserted S-ribs carbon-fiber filaments.

### 4.3. Accuracy Deviations between Experimental and Theoretical Results

The precision analysis of experimental uncertainty for each individual measurement from the experimental results was determined [41] as follows:

(36) $$S_{N_{exp}^{\prime \prime }}={\left\{\sum_{i=1}^{N_{exp}}\frac{{\left(N_{\exp,i}^{\prime \prime }-\bar{N_{\exp,i}^{\prime \prime }}\right)}^{2}}{N_{exp}-1}\right\}}^{1/2}$$

where the mean value of the resulting uncertainty of the experimental measurements was defined by

(37) $$S_{\bar{N_{exp}^{\prime \prime }}}=\frac{S_{N_{exp}^{\prime \prime }}}{\sqrt{N_{exp}}}$$

**Figure 11:**
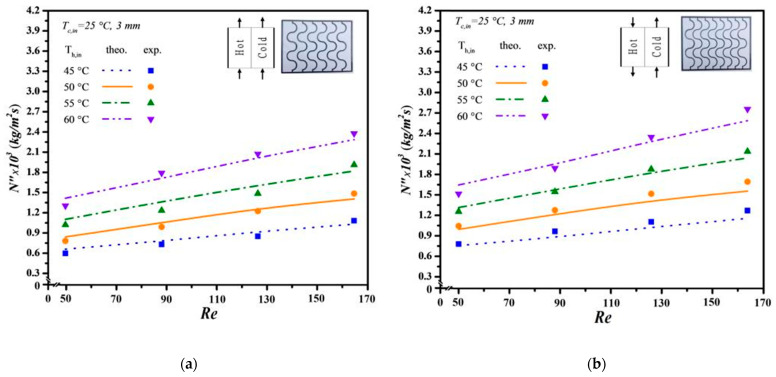
Effect of saline feed temperature and feed flow rate on permeate fluxes (3 mm). (**a**) Cocurrent-flow operations; (**b**) Countercurrent-flow operations.

**Figure 12:**
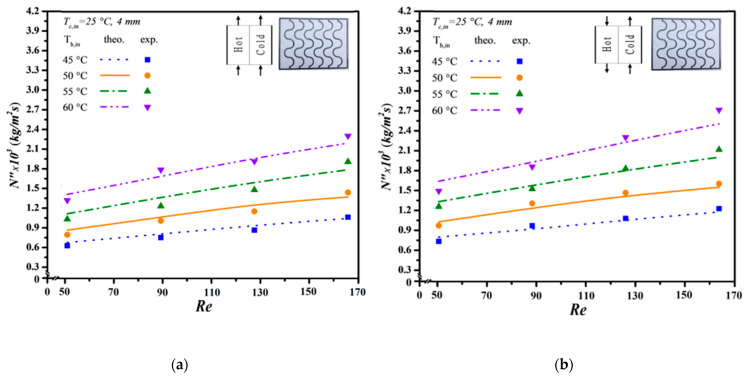
Effect of saline feed temperature and feed flow rate on permeate fluxes (4 mm). (**a**) Cocurrent-flow operations; (**b**) Countercurrent-flow operations.

**Figure 13:**
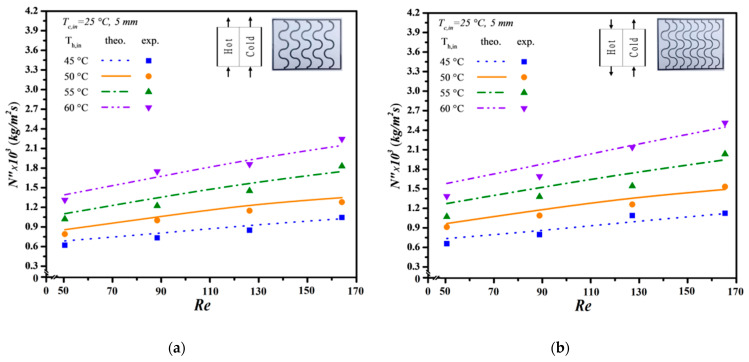
Effect of saline feed temperature and feed flow rate on permeate fluxes (5 mm). (**a**) Cocurrent-flow operations; (**b**) Countercurrent-flow operations.

The mean experimental uncertainty in Figure 11, Figure 12 and Figure 13 ranges within $4.81\times 10^{-3}{\leq S}_{\bar{N_{exp}^{\prime \prime }}}\leq 9.73\times 10^{-3}$. Meanwhile, the accuracy deviation between the experimental results and theoretical predictions was calculated as follows:

(38) $$E=\frac{1}{N_{exp}}\sum_{i=1}^{N_{exp}}\frac{\left|N_{theo,i}^{\prime \prime }-N_{\exp,i}^{\prime \prime }\right|}{N_{theo,i}^{\prime \prime }}$$

where $N_{exp}$, $N_{\exp,i}^{\prime \prime }$, and $N_{theo,i}^{\prime \prime }$ are the number of experimental data, theoretical predictions, and experimental results of permeate flux, respectively. The accuracy deviation of the experimental results from the theoretical predictions is well minimized within $1.83\times 10^{-2}\leq E\leq 9.97\times 10^{-2}$.

Additionally, the predictive capability for permeate flux can be extended to various geometric promoter designs by following the same regression procedure applied to the Nusselt numbers for both modules: one with an empty channel and the others with inserted S-ribs carbon-fiber filaments. In essence, the insertion of S-ribs carbon-fiber turbulence promoters disrupts the thermal boundary layer on the membrane surface, thereby reducing heat-transfer resistance and enhancing permeate flux. The results indicate that higher inlet saline feed temperatures lead to larger $Nu^{p}$ numbers, resulting in a higher heat-transfer rate. Both experimental findings and theoretical predictions of permeate flux were visually presented, utilizing inlet saline feed temperatures and feed flow rates as parameters. This representation is outlined in Figure 11, Figure 12 and Figure 13 for both cocurrent- and countercurrent-flow operations, considering 3 mm, 4 mm, and 5 mm carbon-fiber widths, respectively. The fair consistency and agreement between theoretical predictions and experimental data are evident, providing a robust basis for evidence-based validation, as illustrated in Figure 11, Figure 12 and Figure 13. The order of permeate flux magnitude is observed as 3 mm > 4 mm > 5 mm.

It is noteworthy that a larger permeate flux was achieved at a higher hot saline feed temperature of 60 °C compared to 50 °C. This can be attributed to a greater permeate flux emerging due to a higher saturated vapor pressure gradient between both sides of the membrane. Additionally, a higher hot saline feed flow rate results in an increased permeate flux, facilitated by velocities and vortices that effectively mitigate heat-transfer resistance, especially with smaller carbon-fiber widths impacting the thermal boundary layer. Moreover, the module incorporating 3 mm S-ribs carbon-fiber turbulence promoters in the flowing channel generates more intensive vortices and eddies than those using wider S-ribs carbon-fiber filaments under the same total coverage area. As anticipated, countercurrent-flow operations yield a more significant enhancement in permeate flux compared to cocurrent-flow operations when S-ribs carbon-fiber turbulence promoters are inserted.

A relative increase in permeate flux $I_{N}$ was illustrated by calculating the percentage increment in comparisons between the permeate flux of the module using the empty channel and inserted S-ribs carbon-fiber turbulence promoters as

(39) $$I_{N}=\frac{N_{promoter}^{\prime \prime }-N_{empty}^{\prime \prime }}{N_{empty}^{\prime \prime }}\times 100\%$$

A relative increment of permeate flux $I_{N}$ in the module with inserted S-ribs carbon-fiber turbulence promoters was calculated in comparisons with the module using the empty channel, which were evaluated in terms of the theoretical predictions of permeate flux $N^{\prime \prime }$, as summarized in Table 1 with carbon-fiber width, inlet saline feed temperature and feed flow rate as parameters.

The module with inserted S-ribs carbon-fiber filaments of 3 mm in width exhibits a relative increment in permeate flux of up to 37.77% under countercurrent-flow operations compared to the module using an empty channel. Furthermore, the analysis from Table 1 reveals that the order of device performance for permeate flux enhancement with S-ribs carbon-fiber turbulence promoters increases with higher inlet saline feed temperatures and feed flow rates but decreases with carbon-fiber width. Overall, the insertion of S-ribs carbon-fiber turbulence promoters into the flow channel demonstrates significant potential for substantially augmenting permeate flux in the DCMD module by mitigating the temperature polarization effect.

**Figure 14:**
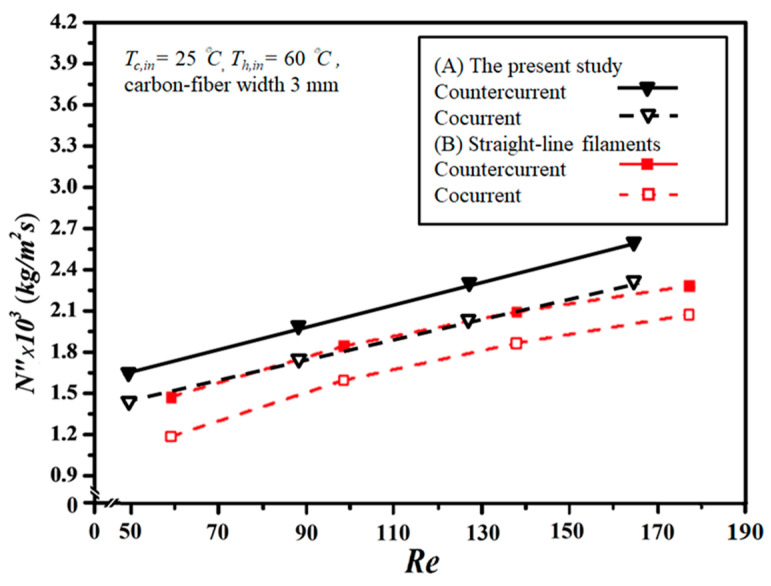
Comparisons of theoretical permeate fluxes of the channels with inserted S-ribs carbon-fiber turbulence promoters and straight-line carbon-fiber filaments [29].

The present work extends the existing study except for inserting S-ribs carbon-fiber turbulence promoters instead of using straight-line carbon-fiber filaments [29]. The impact of the more dominating operational parameter of inlet temperatures was monitored in the DCMD system and the experimental results of the permeate fluxes were collected and weighed to evaluate the improved device performance. Therefore, both inlet and outlet temperatures were measured using thermometer probes for each 5 min interval until the outlet temperatures reached an unchanged steady state, and thus comparisons were made of permeate fluxes under various operation conditions for both modules with and without inserted S-ribs carbon-fiber turbulence promoters. The present study shows a graphical representation of comparisons with the theoretical predictions of permeate fluxes obtained in the present study and straight-line carbon-fiber filaments [29], which illustrates why there is a preference for the present design of inserting S-ribs carbon-fiber turbulence promoters, as shown in Figure 14 for both cocurrent- and countercurrent-flow operations. This is the value and originality of the present study with consideration of the economic viewpoint and technical feasibility.

### 4.4. Energy Consumption Increment

**Figure 15:**
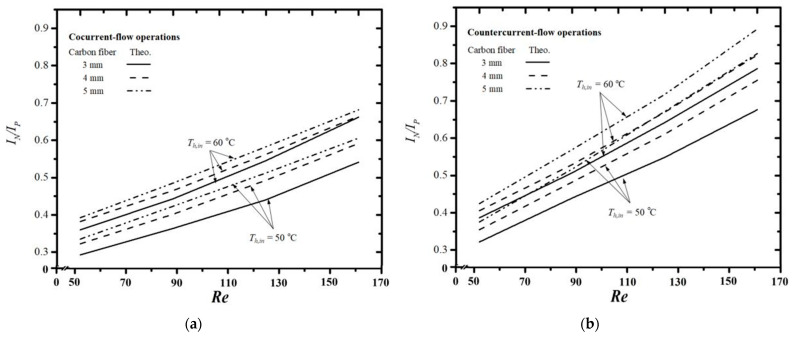
Effect of inlet saline feed temperature and channel designs on the economic feasibility. (**a**) Cocurrent-flow operations; (**b**) Countercurrent-flow operations.

The enhancement of permeate flux is counterbalanced by an increase in energy consumption, constituting trade-offs attributed to heightened turbulent intensity and additional friction losses resulting from the insertion of S-ribs carbon-fiber filaments into the flow channel. An economic consideration on both permeate flux enhancement and power consumption increment for the module with inserted S-ribs carbon-fiber filaments in the present study was also delineated for economic and technical feasibilities, as shown in Figure 15a,b. The impact of hot saline flow rate, inlet saline feed temperature, and carbon-fiber width on the ratio $I_{N}/I_{P}$ is illustrated in Figure 15a,b. This ratio represents the relationship between the power consumption increment and permeate flux enhancement in both cocurrent- and countercurrent-flow operations. An assessment of the effectiveness of membrane turbulence promoters based on economic viewpoint and technical feasibilities was conducted to determine suitable operation and design parameters that balance desirable permeate flux enhancement with undesirable energy consumption increments.

A larger $I_{N}/I_{P}$ value suggests that the increment in energy consumption can compensate for the decrement in permeate flux, owing to the enhancement in convective heat-transfer coefficients increasing permeate flux with the insertion of S-ribs carbon-fiber turbulence promoters. The $I_{N}/I_{P}$ ratio for the channel with wider carbon-fiber filaments exceeds that of the channel with narrower filaments, indicating that adjusting the carbon-fiber width appropriately can achieve more effective permeate flux at the expense of energy consumption. The $I_{N}/I_{P}$ value increases with the hot saline feed temperature and hot saline flow rate but decreases with the S-ribs carbon-fiber width, as revealed in Figure 15a,b. In essence, embedding S-ribs carbon-fiber turbulence promoters in the hot saline feed channel achieves a superior permeate flux enhancement at the cost of a higher friction loss increment under higher inlet saline temperatures. Notably, the values of $I_{N}/I_{P}$ increase with the hot feed flow rate, and larger temperature driving-force gradients in operating countercurrent-flow systems result in a larger value of $I_{N}/I_{P}$.

## 5. Conclusions

The theoretical predictions were calculated and validated by experimental results under various hot feed flow rates, inlet saline temperatures, and various carbon-fiber widths for both cocurrent- and countercurrent-flow operations. Thorough comparisons of permeate flux improvement lead to the following conclusions:

1. Inserting S-ribs carbon-fiber filaments of 3 mm in width into the saline feed flow channel results in relative increases in permeate flux up to a maximum permeate flux improvement of 37.77% under countercurrent-flow operations compared to the module using an empty channel.
2. The results show that permeate flux improvement decreases with the width of carbon-fiber filaments, but the ratio of permeate flux improvement to power consumption increment (say $I_{N}/I_{P}$) increases with the width of carbon-fiber filaments.
3. Permeate flux improvement is more pronounced in countercurrent-flow operations compared to cocurrent-flow operations due to the attainment of a larger temperature gradient.

The correlated equation of the Nusselt number, derived from the theoretical model, proves valuable for designing a more efficient DCMD for membrane desalination applications. While this paper specifically focuses on assessing permeate flux improvement and energy consumption increment by inserting S-ribs carbon-fiber filaments as turbulence promoters into the saline feed channel, further investigation is needed to explore alternative geometric shapes and array configurations of S-ribs carbon-fiber filaments for optimal operation, taking economic feasibility into account.

## Figures and Tables

**Table 1:** Effects of operation conditions and carbon-fiber widths on $N_{\mathrm{theo}}^{\prime \prime }\;$and $I_{N}$, $T_{c,in}=25$ °C.

$T_{h,in}$ (°C)	$Q_{h}$ (*L*/min)	Empty	3 mm		4 mm		5 mm	
		$N_{\mathrm{empty}}^{\prime \prime }\times 10^{3}$	$N_{\mathrm{theo}}^{\prime \prime }\times 10^{3}$	$I_{N}$	$N_{\mathrm{theo}}^{\prime \prime }\times 10^{3}$	$I_{N}$	$N_{\mathrm{theo}}^{\prime \prime }\times 10^{3}$	$I_{N}$
		(kg m^−2^ h^−1^)	(kg m^−2^ h^−1^)	(%)	(kg m^−2^ h^−1^)	(%)	(kg m^−2^ h^−1^)	(%)
50	Cocurrent-flow operations							
	0.3	0.75	0.85	14.06	0.84	12.58	0.83	11.37
	0.5	9.00	1.06	17.46	1.04	15.68	1.03	14.35
	0.7	1.05	1.27	21.14	1.25	19.24	1.23	17.37
	0.9	1.10	1.39	26.00	1.35	23.10	1.33	20.55
60	0.3	1.21	1.42	17.27	1.39	14.92	1.37	13.31
	0.5	1.41	1.71	21.28	1.67	18.16	1.64	16.45
	0.7	1.60	2.02	26.19	1.95	21.94	1.92	19.75
	0.9	1.73	2.28	31.79	2.18	25.95	2.13	23.12
50	Countercurrent-flow operations							
	0.3	0.87	1.00	15.44	0.99	13.82	0.98	12.73
	0.5	1.01	1.22	21.09	1.20	18.81	1.19	17.52
	0.7	1.12	1.42	26.34	1.39	23.84	1.38	22.77
	0.9	1.17	1.55	32.48	1.52	29.49	1.50	28.03
60	0.3	1.39	1.65	18.56	1.61	15.83	1.59	14.39
	0.5	1.57	1.95	24.33	1.89	20.64	1.87	19.30
	0.7	1.75	2.29	30.82	2.21	26.14	2.18	24.31
	0.9	1.88	2.59	37.77	2.48	32.13	2.45	30.27

## Data Availability

Data is contained within the article.

## Abbreviations

A	Cross-sectional area of flow channel (m^2^)
$a_{w}$	Water activity in NaCl solution
C	Friction losses coefficient
$C_{P}$	Heat capacity ($J\;kg^{-1}K^{-1}$)
$c_{k}$	Membrane coefficient based on the Knudsen diffusion model ($\mathrm{mol}\;m^{-2}Pa^{-1}s^{-1}$)
$c_{M}$	Membrane coefficient based on the molecular diffusion model ($\mathrm{mol}\;m^{-2}Pa^{-1}s^{-1}$)
$c_{m}$	Membrane permeation coefficient ($\mathrm{mol}\;m^{-2}Pa^{-1}s^{-1}$)
d	Channel height (m)
$D_{1}$	Carbon-fiber thickness (m)
$D_{m}$	Diffusion coefficient of air and vapor in the membrane ($m^{2}s^{-1}$)
${De}_{h,i}$	Hydraulic equivalent hdiameter of channel (m), i=h, c
${De}_{h,h}^{h}$	The heat-transfer equivalent diameter (m)
E	Accuracy deviation of experimental results from the theoretical predictions
$f_{F,i}$	Fanning friction factor, i=h, c
$h_{c}$	Convective heat-transfer coefficient of cold feed (W $m^{-2}K^{-1}$)
$h_{h}$	Convective heat-transfer coefficient of hot saline feed (W $m^{-2}K^{-1}$)
$h_{h}^{p}$	Convective heat-transfer coefficient of hot saline feed with promoter insertion (W $m^{-2}K^{-1}$)
$H_{i}$	Hydraulic dissipate energy (W), i=h, c
$I_{N}$	Permeate flux relative factor
$I_{p}$	Power consumption relative index
k	Thermal conductivity of water ($Js^{-1}m^{-1}K^{-1}$)
$k_{g}$	Thermal conductivity of gas ($Js^{-1}m^{-1}K^{-1}$)
$k_{m}$	Thermal conductivity of membrane ($Js^{-1}m^{-1}K^{-1}$)
$k_{s}$	Thermal conductivity of solid membrane ($Js^{-1}m^{-1}K^{-1}$)
L	Channel length (m)
$lw_{f,j}$	Friction loss (J kg^−1^), i=h,c
$M_{W}$	Molecular weight of water (kg mol^−1^)
$N_{1}$	Number of carbon-fiber filaments
$N^{\prime \prime }$	Distillate flux ($\mathrm{kg}\;m^{-2}s$)
$\bar{N^{\prime \prime }}$	Average distillate flux ($\mathrm{kg}\;m^{-2}s$)
$N_{exp}$	Number of experimental measurements
${Nu}^{p}$	Enhanced dimensionless Nusselt number
${Nu}_{lam}$	Nusselt number for laminar flow
$P_{m}$	Mean saturated pressure in membrane (Pa)
$P^{sat}$	Saturation vapor pressure (Pa)
Q	Volumetric flow rate (m^3^ s^−1^)
$q^{\prime \prime }$	Heat flux ($J\;m^{-2}s^{-1}$)
$r_{p}$	Membrane pore radius (m)
R	Gas constant (8.314 J mol^−1^ K^−1^)
Re⁡	Reynolds number
$S_{N_{exp}^{\prime \prime }}$	Precision index of an experimental measurements of permeate flux (kg m^−2^ s^−1^)
$S_{\bar{N_{exp}^{\prime \prime }}}$	Mean value of $S_{N_{exp}^{\prime \prime }}$ (kg m^−2^ s^−1^)
$T_{1}$	Membrane surface temperature in the hot saline feed region (°C)
$T_{2}$	Membrane surface temperature in the cold feed region (°C)
$T_{1}^{p}$	Membrane surface temperature with promoter insertion in the hot saline feed region (°C)
$T_{2}^{p}$	Membrane surface temperature with promoter insertion in the cold feed region (°C)
$T_{m}$	Mean temperature in membrane (°C)
$\bar{v}$	Average velocity ($m\;s^{-1}$)
W	Width of channel (m)
$W_{1}$	Carbon-fiber width (m)
$x_{w}$	Liquid mole fraction of water
$x_{\mathrm{NaCl}}$	Mole fraction of NaCl in saline solution
$Y_{w}$	Vapor mole fraction of water
${\left|Y_{m}\right|}_{ln}$	Natural log mean Vapor mole fraction of water in the membrane
z	Axial coordinate along the flow direction (m)
Greek letters	
$\alpha^{p}$	Enhancement factor
$\delta_{m}$	Thickness of membrane (µm)
ε	Membrane porosity
$\eta_{v}$	Gas viscosity ($\mathrm{Ns}\;m^{-2}$)
λ	Latent heat of water ($J\;kg^{-1}$)
μ	Viscosity ($\mathrm{Ns}\;m^{-2}$)
ρ	Density ($\mathrm{kg}\;m^{-3}$)
τ	Membrane tortuosity
$\tau_{temp}$	Temperature polarization coefficients
Subscripts	
1	Membrane surface on hot fluid side
2	Membrane surface on cold fluid side
*c*	Cold feed stream
*h*	Hot feed stream
*cor.*	Correlated results
*empty*	Channel without embedding turbulence promoters
*exp.*	Experimental results
*in*	At the inlet
lam	Laminar flow
*out*	At the outlet
*promoter*	Channel with embedding turbulence promoters
*theo*	Theoretical predictions
